# Molecular characterization of US-like and Asian non-S INDEL strains of porcine epidemic diarrhea virus (PEDV) that circulated in Japan during 2013–2016 and PEDVs collected from recurrent outbreaks

**DOI:** 10.1186/s12917-018-1409-0

**Published:** 2018-03-14

**Authors:** Nguyen Van Diep, Masuo Sueyoshi, Junzo Norimine, Takuya Hirai, Ohnmar Myint, Angeline Ping Ping Teh, Uda Zahli Izzati, Naoyuki Fuke, Ryoji Yamaguchi

**Affiliations:** Department of Veterinary Medicine, Faculty of Agriculture, University of Miyazaki, Gakuen-kibanadai-nishi-1-1, Miyazaki, 889-2192 Japan

**Keywords:** Porcine epidemic diarrhea virus, PEDV, Spike gene, M gene, N gene, Genetic heterogeneity

## Abstract

**Background:**

Since late 2013, porcine epidemic diarrhea virus (PEDV) has reemerged in Japan and caused severe economic losses to the swine industry. Although PEDV vaccines have been used widely, the disease has swept rapidly across the county, and is commonly observed in PED-vaccinated farms, and has recurred in domestic herds. To better understand PEDVs responsible for the reemerging outbreaks in Japan, full-length spike (S), membrane (M), and nucleocapsid (N) genes of 45 PEDVs collected in Japan during 2013–2016, were sequenced and analyzed.

**Results:**

Phylogenetic analysis based on S gene sequences revealed that all the recent field PEDVs were genetically distinct from the classical Japanese strains, and were classified into three genotypes: North American (NA), S INDEL, and Asian non-S INDEL. Our data suggested a possibility that multiple parental PEDV strains were introduced into Japan from abroad at the same time or similar times. The newly identified Japanese strains showed the closest relationship to the US strains. Two sublineages of Japanese strains circulating in Japan were similar to two sublineages identified in the US, suggesting common ancestors for these strains. In comparison with two vaccine strains used in Japan, the field strains had various changes in epitope regions, glycosylation sites, and phosphorylation sites. These substitutions, particularly observed in epitope regions of the S (521, 553, 568, and 570), M (5), and N (123, 252, and 255) proteins, may have affected antigenicity and vaccine efficacy, resulting in an unsuccessful PEDV control. Sequence comparisons between PEDVs collected from primary and secondary outbreaks in three herds revealed that the disease has developed to an endemic stage in which PEDV could persist for nearly two years in the herds or local regions, causing subsequent epidemics.

**Conclusions:**

These results elucidate the genetic characteristics, origin, and molecular epidemiology of PEDVs circulating in Japan, as well as the PEDV strains causing recurrent outbreaks. This study provides a better insight into the PEDVs responsible for recent outbreaks in Japan, and could potentially help to develop measures for controlling and preventing the disease.

**Electronic supplementary material:**

The online version of this article (10.1186/s12917-018-1409-0) contains supplementary material, which is available to authorized users.

## Background

Porcine epidemic diarrhea virus (PEDV) is the etiologic agent of porcine epidemic diarrhea (PED), an acute enteritis disease that is characterized by vomiting and watery diarrhea and usually leads to high morbidity and mortality, especially in piglets [1]. The disease was first recognized in England in 1971 [2], and the prototype virus, which was designated as PEDV CV777, was identified in Belgium [3]. Subsequently, PEDV has been reported in several other European countries, causing only sporadic outbreaks. Since the 1980s, it has been widespread in Asia and has become an economic concern for the swine industry in many Asian countries such as Japan, China, South Korea, Thailand, and Taiwan [1, 4]. In late 2010, new Chinese strains of PEDV were detected. These new strains were clinically more severe than the classical strains, resulting in 80%–100% illness among infected swine herds and a 50%–100% mortality rate among infected suckling piglets [5]. In April 2013, a PEDV outbreak was confirmed in the US for the first time. It swept across more than 30 states in the country, causing deaths of more than 8 million newborn piglets during the one-year-epidemic period, and subsequently spread throughout North America, including Canada and Mexico [6]. Afterward, US-like PED epidemics have been reported in South Korea, Taiwan, Japan, Germany, France, and Belgium [4, 7].

Belonging to the *Coronaviridae* family in the *Nidovirale* order, PEDV has a single-stranded positive-sense RNA genome of approximately 28 kb in size that encodes four structural proteins (S, E, M, and N) and nonstructural proteins (1a, 1ab, and ORF3) [1]. Among the viral proteins, the S glycoprotein is the most diverse [8], and plays a critical role in the process of inducing neutralizing antibodies and binding specific receptors [9]. At least two B cell epitopes (SS2 and SS6) and two regions containing neutralizing epitopes (COE and 2C10) have been identified on this protein [10–12]. The S gene is also associated with growth adaptation in vitro and attenuation of virulence in vivo [13], and was used as an important component in studies for understanding genetic relatedness among PEDV isolates, the epidemiological status, and vaccine development [14, 15]. The M protein is the most abundant component of viral protein in the envelope, and is responsible for morphogenesis, assembly, and budding [16]. The N protein of coronavirus interacts with viral genomic RNA, serving as the critical basis for the helical nucleocapsid during the viral assembly [17]. Two antigenic epitopes (NEP-D4 and NEP-D6) have been identified in the N protein of PEDV [18].

In Japan, the PED-like disease was observed from late 1982 to early 1983, but was not reported again until the outbreaks that occurred between September 1993 and June 1994 [19]. In an epidemic of diarrhea caused by a PEDV infection in 1996, more than 39,000 suckling pigs died [20]. Afterward, there were no PED cases reported from 2006 until the first half of 2013 [21, 22]. PED outbreaks first reemerged in October 2013 from a southern location (Okinawa Island) and spread rapidly throughout the country. Until May 2016, PEDV infections were reported from more than 1000 farms in 39 of 47 prefectures; the infections affected over 1.5 million pigs and led to the death of half a million pigs, according to the Ministry of Agriculture, Forestry, and Fisheries of Japan (http://www.maff.go.jp). Currently, PEDV infections and piglet mortality has decreased significantly. However, PED still occurred and frequently recurred in pig farms in Japan. Therefore, better insight into the causes for the reemergence of PED outbreaks is required for more efficient control and prevention of the disease. Toward this goal, we sequenced and analyzed the full-length S, M, and N genes of the Japanese field PEDVs in comparison with those of vaccine strains used in Japan, classical Japanese PEDV strains, and isolates from other countries.

## Methods

### Sample collection, RNA extraction, and PEDV detection

Small intestine or stool specimens were obtained from suckling piglets, post-weaning piglets, and sows presenting acute watery diarrhea at pig farms in Japan, between December 2013 and February 2016. Intestinal samples were collected from dead piglets, and fecal samples were non-invasively collected immediately after excretion. Thus, no aggressive actions toward the pigs were carried out for sampling purpose. Samples of two major vaccine strains that have been employed in Japan, P5-V (Nisseiken Co., Ltd) and 96-P4C6 (Kaketsuken Co., Ltd., Japan), were collected from commercial vaccine bottles used in the swine farms. Sample preparation, RNA extraction, and PEDV detection were performed as previously described [23]. Japanese field samples may contain both PEDV variants with large deletions in the 5′-terminus of the S gene and PEDV strains with an intact S gene [24]. Due to the complexity in discriminating, isolating, and individually sequencing PEDV strains, only specimens containing PEDVs with an intact S gene were used in our study. Finally, 45 PEDV-positive field samples were collected from 37 farms, located in five different prefectures in Japan. These samples, along with the two vaccine samples described above, were sequenced to determine the full-length sequence of the S, M, and N genes (Table 1). All these field PEDVs were confirmed to possess an intact 5′-terminus of the S gene, showing only the expected prominent single DNA band from PCR products on a 1.2% agarose gel [24].

**Table 1:** Forty-five Japanese field PEDVs and two vaccine strains were used in this study

No	Strain	Age group	Collection time	Geographic origin	Accession No of genes		
					S gene	M gene	N gene
1	14JM-01	Suckling	2014/Mar	Miyazaki	KY619734	KY619830	KY619781
2	14JM-07	Post-weaning	2014/Apr	Miyazaki	KY619735		KY619782
3	14JM-40	Suckling	2014/Apr	Hokkaido	KY619736		KY619783
4	14JM-118	Suckling	2014/Mar	Miyazaki	KY619737		KY619784
5	14JM-119	Suckling	2014/Apr	Miyazaki	KY619738		KY619785
6	14JM-123	Suckling	2014/Mar	Miyazaki	KY619739		KY619786
7	14JM-126	Suckling	2014/Mar	Miyazaki	KY619740		KY619787
8	13JM-127	Suckling	2013/Dec	Miyazaki	KY619741	KY619831	KY619788
9	13JM-128	Suckling	2013/Dec	Miyazaki	KY619742	KY619832	KY619789
10	14JM-138	Suckling	2014/Mar	Miyazaki	KY619743		KY619790
11	14JM-139	Suckling	2014/Mar	Miyazaki	KY619744		KY619791
12	14JM-140	Suckling	2014/Mar	Miyazaki	KY619745		KY619792
13	14JM-142	Suckling	2014/Mar	Miyazaki	KY619746		KY619793
14	14JM-143	Suckling	2014/Feb	Miyazaki	KY619747		KY619794
15	14JM-144	Suckling	2014/Mar	Miyazaki	KY619748	KY619833	KY619795
16	14JM-146	Suckling	2014/Apr	Miyazaki	KY619749		KY619796
17	14JM-147	Suckling	2014/May	Miyazaki	KY619750		KY619797
18	14JM-150	Suckling	2014/Mar	Miyazaki	KY619751		KY619798
19	14JM-152	Suckling	2014/Mar	Miyazaki	KY619752		KY619799
20	14JM-157	Suckling	2014/May	Aichi	KY619753		KY619800
21	14JM-168 ^farm 1^	Suckling	2014/May	Aomori	KY619754		KY619801
22	14JM-179 ^farm 2^	Suckling	2014/May	Miyazaki	KY619755		KY619802
23	14JM-181 ^farm 2^	Suckling	2014/May	Miyazaki	KY619756		KY619803
24	14JM-199 ^farm 3^	Suckling	2014/May	Miyazaki	KY619757		KY619804
25	14JM-200 ^farm 3^	Suckling	2014/May	Miyazaki	KY619758		KY619805
26	14JM-205	Suckling	2014/Jun	Hokkaido	KY619759		KY619806
27	14JM-208	Suckling	2014/Jun	Aichi	KY619760	KY619834	KY619807
28	14JM-210	Sow	2014/Jul	Aichi	KY619761		KY619808
29	14JM-216	Sow	2014/Jul	Aichi	KY619762	KY619835	KY619809
30	14JM-226	Suckling	2014/Jul	Kagoshima	KY619763		KY619810
31	14JM-236	Suckling	2014/Jul	Miyazaki	KY619764	KY619837	KY619811
32	14JM-242	Suckling	2014/May	Miyazaki	KY619765		KY619812
33	14JM-248	Suckling	2014/Jane	Miyazaki	KY619766		KY619813
34	14JM-268	Suckling	2014/May	Miyazaki	KY619767		KY619814
35	13JM-291	Suckling	2013/Dec	Kagoshima	KY619768		KY619815
36	13JM-293	Suckling	2013/Dec	Kagoshima	KY619769		KY619816
37	14JM-297	Suckling	2014/Apr	Aichi	KY619770		KY619817
38	14JM-311	Suckling	2014/Apr	Aichi	KY619771		KY619818
39	15JM-315	Suckling	2015/Jun	Aichi	KY619772		KY619819
40	16JM-319 ^farm 3^	Suckling	2016/Feb	Miyazaki	KY619773	KY619836	KY619820
41	16JM-323 ^farm 3^	Suckling	2016/Feb	Miyazaki	KY619774		KY619821
42	16JM-325 ^farm 2^	Suckling	2016/Feb	Miyazaki	KY619775		KY619822
43	16JM-326 ^farm 2^	Suckling	2016/Feb	Miyazaki	KY619776		KY619823
44	16JM-334 ^farm 1^	Suckling	2016/Feb	Aomori	KY619777	KY619838	KY619824
45	16JM-339 ^farm 1^	Suckling	2016/Feb	Aomori	KY619778	KY619839	KY619825
46	96P4C6	Nisseiken Co	2013/Dec		KY619779	KY619828	KY619827
47	P5-V	Kakatsuken Co	2014/Apr		KY619780	KY619829	KY619826

- The following Japanese field strains have the same S gene sequences: 14JM-127 and 14JM-311; 14JM-01, 14JM-119, and 14JM123; 14JM-138, 14JM-142, and 14JM-150; 14JM-208 and14JM-216; 14JM-146, 14JM-147, and 14JM-199; 16JM-334 and 16JM-339; 16JM-319, 16JM-323, and 16JM-325

-The following Japanese field strains have the same M gene sequences: 14JM-01, 14JM-07, 14JM-40, 14JM-118, 14JM-119, 14JM-123, 14JM-126, 14JM-138, 14JM-139, 14JM-140, 14JM-142, 14JM-143, 14JM-146, 14JM-147, 14JM-150, 14JM-152, 14JM-157, 14JM-168, 14JM-179, 14JM-181, 14JM-199, 14JM-200, 14JM-205, 14JM-210, 14JM-226, 14JM-242, 14JM-248, 14JM-268, 13JM-291, 13JM-293, 14JM-297, 14JM-311, and 15JM-315; 16JM-319, 16JM-323, 16JM-325, and 16JM-326; 13JM-127 and 13JM-128; 14JM-208 and 14JM-216; 16JM-334 and 16JM-339

- The following Japanese field strains have the same N gene sequences: 13JM-127, 13JM-128, 14JM-142, 14JM-143, 14JM-157, 14JM-208, 14JM-216, 14JM-248, and 13JM-291; 14JM-01 and 14JM-119; 14JM-07, 14JM-126, and 14JM-123; 14JM-138, 14JM-150, and 14JM-226; 14JM-139, 14JM-152, and 14JM-242; 14JM-146, 14JM-147,14JM-179, 14JM181, 14JM-199, 14JM-200, 14JM-236, and 14JM-311; 14JM-268 and 13JM-293; 16JM-319 and 16JM-323; 16JM-334 and 16JM-339; 16JM-325 and 16JM-326

### Sequencing of S, M, and N genes

After producing cDNA, as previously described [23], the full-length S gene of PEDV was amplified using the primer pair FS-F/FS-R (Table 2) and KOD FX Kit (Toyobo Co., Japan). The PCR products were used as templates for nested PCR reactions that amplified five DNA fragments spanning the entire S gene using 5 primer sets (CS1-CS5), as previously reported [24]. The M and N genes of the PEDV were also amplified from the cDNA using two newly designed primer sets, fMF/fMR and fNF/fNR (Table 2). EmeraldAmp MAX PCR Master Mix kit (Takara Bio, Japan) was used for PCR amplifications of the CS1-CS5 fragments, as well as the M and N genes. The amplified PCR products were purified using a FastGene Gel/PCR Extraction Kit (NIPPON Genetics Co., Ltd., Japan) according to the manufacturer’s protocol. All sequencing reactions were carried out in duplicate, and sequences were determined in both directions using BigDye® Terminator v3.1 Cycle Sequencing Kits (Applied Biosystems, CA, USA). Products were analyzed using ABI PRISM 3130xl Genetic Analyzers (Applied Biosystems).

**Table 2:** Primers used in this study

Primer	Nucleotide sequence (5′-3′)	Target gene (fragment size, bp)	Position^a^
FS-F	TCCATTAGTGATGTTGTGTTAGG	Full-length S gene (4371)	20,530–24,900
FS-R	ACTACATTRAGCTCCAACTC		
fMF	CTTGTCACCGGTTGTGTAATAG	Full-length M gene (826)	25,567–26,392
fMR	CTGACAGAAGCCATAAAGTTTCTG		
fNF	ACTGGTTGGGCTTTCTATGTC	Full-length N gene (1507)	26,263–27,769
fNR	CTCAGTAATAACAGTGTAATGGCAC		

^a^Numbers correspond to positions within the Colorado/USA/2013 genome

### Sequence analysis

Nucleotide (nt) and deduced amino acid (aa) sequences were edited, assembled, and aligned using Geneious v9.1.6 software (http://www.geneious.com), and percentage sequence divergences at the nt and aa levels were further calculated by using the same software. The obtained nt sequences were deposited in GenBank under the following accession numbers: KY619734–KY619839 as shown in Table 1. Unrooted phylogenetic trees were constructed using molecular evolutionary genetics analysis (MEGA) software, version 6.06 [25], with the maximum likelihood method and bootstrap tests of 1000 replicates. The best-fit nt substitution models for analysis were assessed. Phylogenetic trees based on the nt sequences of the full-length S and N genes were generated using the Tamura-Nei substitution model with a discrete gamma distribution (TN93 + G). A phylogenetic tree based on the nt sequences of the M gene was created using the Kimura 2-parameter substitution method with a discrete gamma distribution (K2 + G). N-glycosylation sites were predicted using a service available on http://www.cbs.dtu.dk/services/NetNGlyc. Phosphorylation sites were predicted using the NetPhos 3.1 server (http://www.cbs.dtu.dk/services/NetPhos) and NetPhosBac 1.0 server (http://www.cbs.dtu.dk/services/NetPhosBac-1.0). Prediction of antigenic regions was performed using the EMBOSS protein analysis tool [26] integrated into the Geneious software.

## Results

### Analysis of the S gene

#### Phylogenetic and sequence analyses of the S gene

**Fig. 1:**
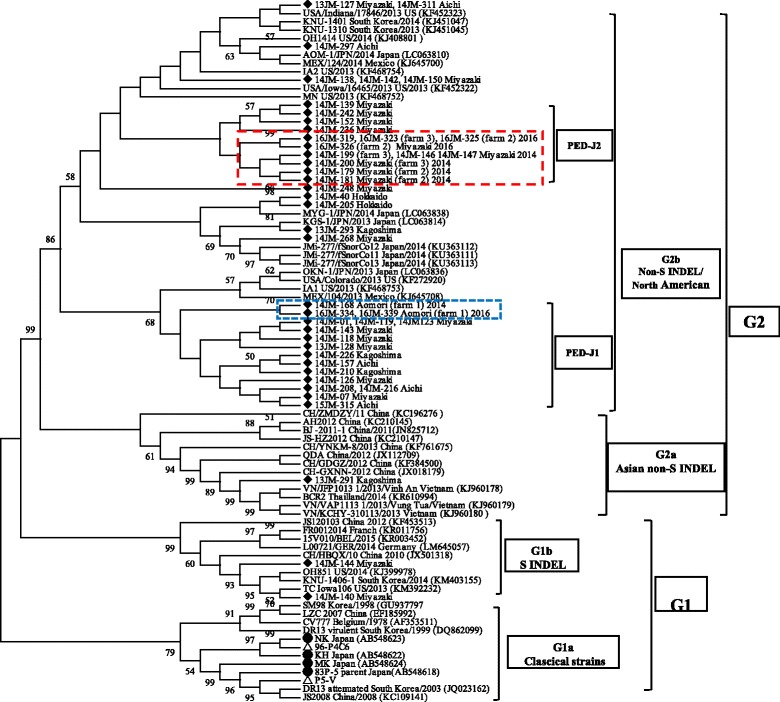
Phylogenetic tree of PEDVs based on the nucleotide sequences of the entire S gene. The tree was constructed by the maximum likelihood method using the Tamura-Nei substitution model with a discrete gamma distribution in the MEGA v.6.05 program. Numbers at nodes represent the percentage of 1000 bootstrap replicates (values < 50 are not shown). Names of strains, countries and years of isolation, and GenBank accession numbers are shown. The Japanese field PEDV strains identified in this study are marked by solid diamond symbols and accompanied by the corresponding prefectures where they were collected. The classical Japanese strains identified prior to 2013 are marked by solid round symbols and the vaccine strains being used in Japan are marked by hollow triangle symbols. PEDV strains collected from farm 1 (14JM-168, 16JM334, and 16JM-339) were marked in a blue box; strains collected from farms 2 and 3 (14JM-179, 14JM-181, 14JM-199, 14JM-200, 16JM-319, 16JM-323, 16JM-325, 16JM-326 are marked in a red box

Identical nt sequences of the S gene were distinguished and excluded, resulting in the identification of 34 individual sequences from the 45 total collected field strains. Phylogenetic analysis based on the S gene sequences from the Japanese strains and reference strains identified from various countries revealed two major clusters: genogroup G1 divided into subgroups G1a and G1b, and genogroup G2 divided into subgroups G2a and G2b (Fig. 1).

All 45 Japanese field PEDVs in the present study fell into 3 subgroups G2a, G2b, and G1b. The most dominant strains belonged to G2b, which comprised 42 of 45 current Japanese strains, along with the highly virulent (North American type) PEDVs isolated in the US, Canada, Mexico, South Korea, and Taiwan. Two PEDV strains (14JM-140 and 14JM-144) were clustered into G1b, which included the new S INDEL strains that have been detected since 2013 in the US, Germany, France, Belgium, South Korea, Japan, and China. Isolate 13JM-291 fell into G2a, which included PEDV strains identified from PEDV outbreaks that occurred after 2010 in Vietnam, Thailand, and China. The two vaccine strains (P5-V and 96-P4C6) clustered into the G1a subgroup, which primarily consisted of classical strains, such as CV777, KH, NK, LZC, SM98, and DR13 parent.

Pairwise alignment of the 45 field PEDVs in this study revealed that their S genes were heterogeneous. The S gene nt (aa) sequences had 94.36%–100% (94.45%–100%) identity with each other, but only 93.24%–95.91% (92.01%–96.17%) identity with the two Japanese vaccines. Most of the Japanese PEDVs belonging to G2b had 99.16%–100% nt among themselves, and shared a high (98.27%–100%) nt identity with other highly virulent PEDVs. Interestingly, 13JM-127 from the first PED outbreak in Miyazaki prefecture (South Japan, December 2013) shared the same sequence with 14JM-311 (Aichi prefecture, north-central Japan, April 2014) and US strains, such as USA/Indiana/17846/2013 (from the first reported PED case in the US) [27], USA/Colorado/2013, OH1414, and IA1. No relatedness were found between the two farms from where 13JM-127 and 14JM-311 were collected. Additionally, 14JM-01 has 100% nt sequence identity with two other US strains (NPL-Pedy/2013 US and USA/Minnesota127/2014) and a Canadian strain (ON-007).

Of the 45 field PEDVs, 14JM-01 from Miyazaki and 16 other strains from Aomori, Aichi, Kagoshima, and Miyazaki were grouped into a monophyletic branch designated as PED-J1, and they shared high nt and aa sequence identities (99.69%–100% and 99.35%–100%, respectively) with each other. Another subclade, designated as PED-J2, including 14 Japanese strains collected in Miyazaki were also clustered into a segregated branch as shown in Fig. 1. The strains 14JM-140 and 14JM-144 showed 98.99%–99.92% nt sequence identity with other S INDEL strains within G1b. The S gene of 14JM-144 had the highest (99.93%) nt sequence identity with OH851 which was the first reported S INDEL strain in the US. The S gene from 13JM291 shared 97.64%–99.35% nt sequence identity to those of other G2a strains, with the highest nt identity (99.3%) to those of VN/VAP1113_1/2013/VungTua/Vietnam and VN/KCHY_310113/2013/KhoaiChau/HungYen from Vietnam, BCR2 from Thailand, and CH/GXNN/2012 from China.

The sequence data revealed that S genes from the Japanese field PEDVs are of 4152–4161 nt long, and encode proteins with 1381–1386 aa residues. This consequence was due to the presence of several deletions or insertions that accumulated primarily in the N-terminus of the S protein. Multiple alignments of the S gene demonstrated that 14JM-140 and 14JM-144 exhibited insertions and deletions (1-nt, 11-nt, and 3-nt deletions at positions 167, 176, and 416, respectively, and a 6-nt insertion between positions 474 and 475), which are typical for S INDEL strains [28, 29].

**Fig. 2:**
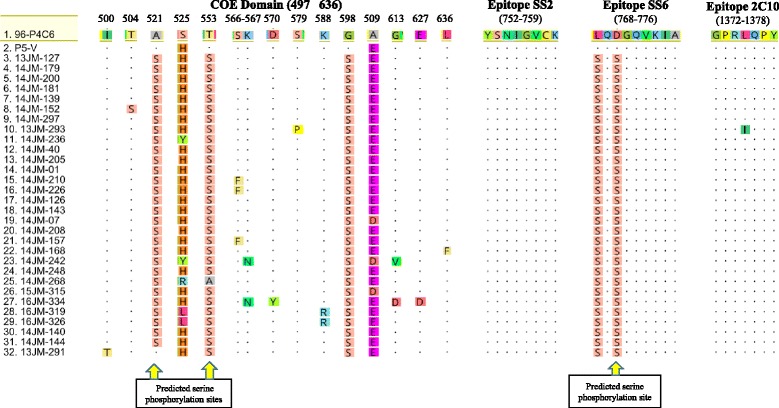
Amino acid substitutions in the S protein epitope regions of the field and vaccine strains. The twelve substitutions between the Japanese field strains and vaccine strains were I500T, T504S, H/S525Y/R/L, S566F, K567 N, D570Y, S579P, K588R, A/E509D, V613D/V, E627D, and L636F. Predicted serine phosphorylation was occurred at positions 521, 553, and 770

Compared to the two vaccine strains, all Japanese field PEDVs were highly conserved in epitopes 2C10 and SS2, except for strain 13JM-293 that had an aa substitution (L1375I) within 2C10 (Fig. 2). Additionally, compared to the vaccine strains, all the Japanese field PEDVs had two aa changes (L768S and D770S) in the epitope SS2, and three serine substitutions (A521S, T553S, and G598S) in the neutralizing domain COE (excluding 13JM291 and 14JM-268). Notably, the aa substitutions at positions 521 and 553 resulted in predictable phosphorylation sites in the S protein of the Japanese field strains. Furthermore, different amino acids were found at the 12 sites within the COE domain (Fig. 2).

Regarding the prediction of high-specificity N-glycosylation sites in the S protein, most Japanese strains belonging to G2b exhibited eight N-glycosylation sites over the entire S protein (Additional file 1: Table S1). 14JM-268 had one additional N-glycosylation site at residue 557, and 14JM-242 possessed an additionally unique N-glycosylation site at position 382 of the aa chain. Two Japanese S INDEL strains (14JM-140 and 14JM-144) had seven N-glycosylation sites, while 13JM-291 had eight N-glycosylation sites. Compared with the vaccine strain, 96-P4C6, most of the Japanese non-S INDEL strains in G2b lost three N-glycosylation sites (at 132, 557, and 1233) and gained another N-glycosylation site (at 60). Compared with P-5 V, these G2b Japanese strains lost three N-glycosylation sites (at 117, 132, and 515) and gained four other N-glycosylation sites (at 60, 119, 325, and 1262).

### Analysis of the M gene

**Fig. 3:**
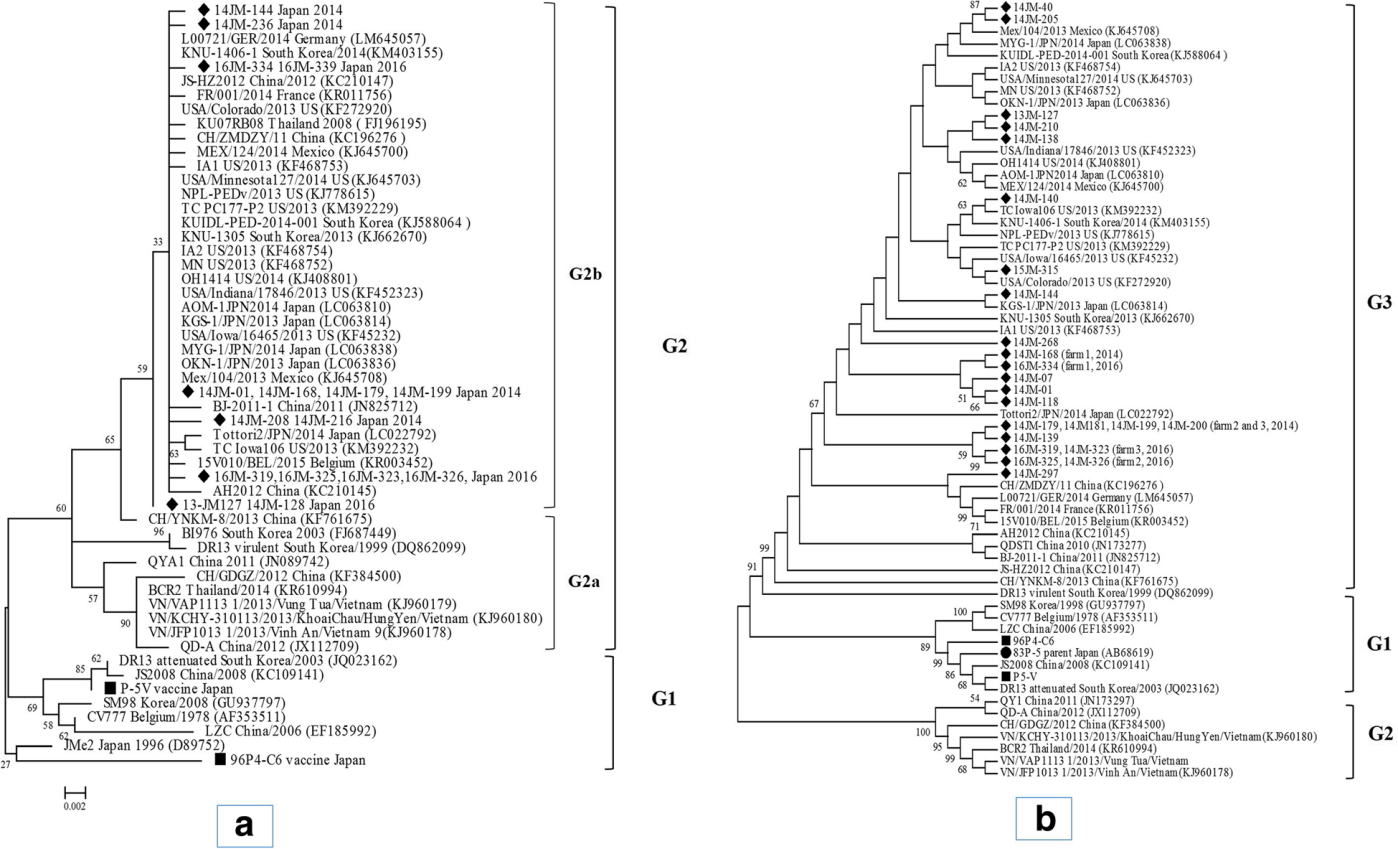
Phylogenetic trees of PEDVs based on nucleotide sequences of entire M and N genes. Phylogenetic trees based on the M gene (**a**) and the N gene (**b**) were constructed using the maximum likelihood method in the MEGA program. Numbers at nodes represent the percentage of 1000 bootstrap replicates. The scale bars indicate the number of nucleotide substitutions per site. Names of strains, countries and years of isolation, and GenBank accession numbers are shown. The Japanese field PEDV strains identified in this study are marked by solid diamond symbols and the vaccine strains used in Japan are marked by solid square symbols

The M gene sequences from all the Japanese field PEDVs and the two vaccine strains consisted of 681 nucleotides, encoding a protein of 226 aa. Identical nt sequences were distinguished and excluded, resulting in the identification of seven unique sequences from the 45 total collected PEDVs (Table 2). A phylogenetic tree based on the M gene of the Japanese strains and reference strains revealed that all the sequences were divided into 2 groups (G1 and G2); G2 was further divided into two subgroups G2a and G2b (Fig. 3a). All the current Japanese PEDVs fell into G2, and showed the closest relatedness to the NA strains and emerging strains identified in Europe, South Korea, and China. These field PEDVs were phylogenetically distant from the vaccine strains and a classical Japanese strain (JMe2) of G1. The M genes of field PEDVs had 99.56%–100% nt identity to each other and 97.5%–98.24% nt identity with the vaccine strains.

The B cell epitope, WAFYVR [30], located at position 195–200 of the M protein, was conserved in all field and vaccine strains. Compared with the vaccine strains, all Japanese field strains had an aa substitution (A214S) belonging to an antigenic region (aa 205–223), as predicted by the EMBOSS protein analysis tool (Additional file 2: Figure S1A). Strain 14JM-236 had a different aa (S213 N) leading to the loss of a predicted serine phosphorylation site. In another predicted epitope region (aa 5–15), the field PEDVs had one different aa (V12I) compared to P-5 V, and two aa substitutions (F5S and Q13E) compared to 96-P4C6. These substitutions (F5S) resulted in gaining a predicted high-specificity N-glycosylation site.

### Analysis of the N gene

The N gene of all the field PEDVs and vaccine strains was 1326 nt in length encoding a protein of 441 aa. Identical nt sequences of the N gene were distinguished and excluded, resulting in the identification of 19 unique sequences. Phylogenetic analysis based on the N genes from the Japanese strains and reference strains revealed the classification of PEDV strains into three groups: G1, G2, and G3 (Fig. 3b). All Japanese field strains were closely clustered together into subgroup G3 with other US and US-like strains. The Japanese field PEDVs shared 98.72%–100% N gene nt sequence identity to each other and 95.70%–96.76% to the vaccine strains. Strain 13-JM127 from the first outbreak in Miyazaki had 100% nt identity to eight other field strains collected in Miyazaki, Aichi, and Kagoshima, and this is likely to be the most dominant sequence of N gene among the field strains. Compared to the vaccine strains, all the field strains in the present study had 9 aa substitutions (Additional file 2: Figure S1B); the substitution K123 N resulted in forming a predicted N-glycosylation site on the N protein from the field strains, while the aa substitutions A142T and N255S observed in field strains formed two predicted phosphorylation sites. Of note, the substitutions K123 N and N255S belong to the reported antigenic epitopes NEP-D4 (aa 18–133) and NEP-D6 (aa 252–262), respectively [18].

### Comparison between strains collected from primary and secondary outbreaks

There were three swine farms where PED outbreaks first occurred in 2014, and then recurred in 2016, as shown in Table 3. Farm 1 is located in Aomori prefecture, while farms 2 and 3 are located in the same town in Miyazaki prefecture. Phylogenetic analysis based on the S gene sequences revealed that three PEDV strains (14JM-168, 16JM-334, and 16JM-339) collected from two different outbreaks on farm 1 were closest to each other and segregated into a distinct minor branch. PEDVs from the primary PED outbreaks on farm 2 (14JM-179, 14JM-181) and farm 3 (14JM199 and 14JM-200) grouped closest together, along with two other strains (14JM-146 and 14JM-147) from different pig farms in Miyazaki prefecture in 2014. Likewise, strains from recurrent outbreaks on farm 2 (16JM-325 and 16JM-326) and farm 3 (16JM-319, 16JM-323) were also grouped into a monophyletic branch. All the PEDV strains collected from the primary and recurrent outbreaks on farms 2 and 3 were clustered into the minor group PED-J2, along with several strains collected from PED outbreaks that occurred on other pig farms in Miyazaki in 2014. Similar phylogenetic relatedness among PEDVs obtained from primary and recurrent outbreaks was observed, based on the phylogenetic tree of the N gene.

**Table 3:** Information of primary and recurrent PED outbreaks occurred in the three pig farms in this study

	Farm 1		Farm 2		Farm 3	
	Primary outbreak	Recurrent outbreak	Primary outbreak	Recurrent outbreak	Primary outbreak	Recurrent outbreak
Outbreak time	May-2014	Feb-2016	May-2014	Feb-2016	May-2014	Feb-2016
Scale of farm	2000 sows	2000 sows	580 sows	600 sows	700 sows	700 sows
Vaccination	No	Yes	Yes	Yes	Yes	Yes
Morbidity (%)	100	60	100	70	100	70
Mortality (%)	100^a^	50^b^	70^a^	30^a^	70^a^	30^a^
Sample	14JM-168	16JM-334, 16JM-339	14JM-179, 14JM-181	16JM-325, 16JM-326	14JM-199, 14JM-200	16JM-319, 16JM-323

^a^Mortality rate of piglets less than two weeks-old; ^b^Mortality rate of the piglets less than one-week-old

All the information for mortality and morbidity was kindly supplied by the veterinarians responsible for these farms

On farm 1, the S, M, and N genes of PEDVs identified from the recurrent outbreak (16JM-334 and 16JM-339) shared the highest (99.66%, 100%, and 99.55% respectively) nt sequence identities to a strain (14JM-168) collected from the primary outbreak. The S genes of PEDVs from the primary outbreak on farms 2 and 3 had high (99.92%–99.98%) nt identity to each other. These strains also had identical sequences for the M and N genes. In the secondary outbreaks, PEDVs from farms 2 and 3 also shared highly homologous genes. The S and M genes of two strains (16JM-319 and 16JM-323) from farm 3 shared 100% nt sequence identities with those of a strain (16JM-325) from farm 2.

Compared with the strain collected from the primary outbreak on farm 1, two strains from the recurrent outbreak had 11 aa substitutions in their S proteins (Additional file 3: Figure S2). Notably, the substitution S722 N lead to the loss of a predicted serine phosphorylation site. These recurrent strains also had two aa substitutions (R166S and T413 N) in the N protein; the R166S substitution formed a predicted serine phosphorylation site. Comparison to PEDVs from the primary outbreak on farms 2 and 3, PEDVs from the recurrent outbreaks had 13 aa substitutions in the S proteins, in addition to a single aa change (A42V) in the M protein and two aa changes (L18I and S31F) in the N protein. Notably, the substitutions L18I and S31F occurred in the reported epitope NEP-D4 (18–133) of the N protein.

## Discussion

Since late 2013, massive PED outbreaks have recurred in Japan causing tremendous financial losses in the swine industry. Despite the nationwide use of available attenuated vaccines developed decades ago [21], PEDV has rapidly swept across the county. Our sequencing data revealed that the prevailing Japanese PEDVs are genetically heterogeneous and can be classified into 3 genotypes: NA, S INDEL, and Asian non-S INDEL. Both the NA and S INDEL types, which have been identified in recent studies in Japan [21–23, 31] were also responsible for recent outbreaks in the US and South Korea. In our study, besides the two previously reported PEDV types, we identified another type of Japanese PEDV (designated as Asian non-S INDEL) that is closely related to the Vietnamese, Thai, and Chinese strains. The Asian non-S INDEL type was collected from an early PED epidemic that occurred in southern Japan where the recent PED pandemic began spreading. Moreover, the Asian non-S INDEL type appeared at a similar time to the appearance of the NA and S INDEL types in Japan [21]. Together with the absence of PED cases from 2007 to 2012 in Japan [21, 22], we speculated a possibility that the three types of Japanese PEDV originated from one source and emerged at the same or similar time in the southern region of Japan before spreading across the country*.*

The result of this study revealed that all the field PEDV strains (except 13JM-291) were closely related to emerging strains identified in the US, South Korea, Taiwan, and in European countries. Of note, the Japanese PEDVs were genetically most closely related to the US strains; some even had identical S gene. Since the recent PED pandemics in Japan started to reemerge six months after those in the US, the close genetic relationship indicated that the current Japanese PEDV might have been introduced directly from the US, or both the US and Japanese PEDVs were derived from one source of origin.

Since April 2013, the NA type of PEDV has emerged in the US. The NA strains were identified subsequently in South Korea, Taiwan, and Japan from late 2013. Another PEDV type possessing typical insertions and deletions in the N-terminus of the S gene when compared to the prototype NA type was first discovered in Ohio in the US [29]. This PEDV type, therefore, was designated as S INDEL. The S INDEL PEDVs were also identified later in recent outbreaks in Japan, South Korea, Canada, Belgium, France, Germany, Portugal, Slovenia, and the Netherlands [4]. Both highly virulent and S INDEL PEDVs prevailing in the US are supposed to be derived from Chinese PEDVs as a result of recombinant events [4, 28, 32]. In accordance with these findings, our sequence analysis showed that the S INDEL strains, such as CH/HBQX/10 and JS120103, had already appeared in Chinese PED outbreaks during 2010–2012 [33, 34]. With the prevalence of both NA and S INDEL strains in the US, South Korea, and Japan since 2013, we suggested that there are two major possibilities of origin for the reemerging Japanese PEDV strains. First, US-like strains have been directly transmitted to Japan from the US or South Korea. Second, the same source of the US strains could have introduced the PEDV into Japan, from China, shortly after the US outbreaks. However, our study revealed the prevalence of the Asian none-S IDEL PEDV strain in Japan, which has not been reported in the US or South Korea. Thus, we incline toward the possibility that multiple parent PEDV strains have been introduced into Japan, from China or China’s neighboring countries (such as the Southeast Asian region), causing the recent PED pandemic. Further investigation and surveillance are required to specifically identify the source of origin for the reemerging Japanese PEDVs.

Sixteen PEDV strains from four different prefectures including Miyazaki, Kagoshima, Aomori, and Aichi formed a well-supported subclade (PED-J1). Fourteen PEDV strains from various farms in Miyazaki prefecture were also grouped into a distinct subclade (PED-J2). These results suggested that strains of those Japanese sublineages (PED-J1 and PED-J2) may derive from two common ancestral PEDVs whose progeny have spread in the regions. Two genetic sublineages of US strains, namely IA1-Co/13 and MN-IA2 [32], were identified from the US outbreaks in 2013. Notably, the Japanese PEDV of the Japanese sublineage PED-J1 in this study showed a close relationship with the US sublineage IA1-Co/13, and PEDVs of the Japanese sublineage PED-J2 showed close relatedness with the US sublineage MN-IA2. This data revealed the common pattern of genetic diversity as well as the close relationship between the Japanese strains and the US strains, suggesting a common ancestor for these strains.

In our previous study [23], by sequencing a partial S gene containing a neutralizing epitope region (COE domain), we suggested that 14JM-140 may belong to the S INDEL type that has been circulating in Japan together with the highly virulent US-like strains. In this study, by sequencing the full-length S gene, we confirmed 14JM-140 to be an S INDEL strain. We also identified another strain that belonged to the S INDEL type, 14JM-144, although it was not predicted to be an S INDEL PEDV in the previous study. This discrepancy arose due to the region of the partial S gene utilized in the previous report that lack the typical insertions and deletions of the S INDEL type. Our data revealed the circulation of S INDEL strains in PED outbreaks occurring in Japan, but these strains compose only a minor population of the reemerging Japanese PEDVs. These results are consistent with two recent reports of Japanese PEDV [21, 22].

Phosphorylation of viral proteins is a reversible post-translational modification that can have major impacts on viral infection, replication, and cytotoxicity in a host cell [35]. For infectious bronchitis virus, belonging to the coronaviruses, the phosphorylation of the nucleocapsid protein has a dramatic influence on RNA binding capability and the recognition of viral RNA from non-viral RNA in the process of viral replication [36]. Along with phosphorylation, glycosylation is another post-translational modification that often strongly affects the protein functions of viruses. N-glycosylation sites in the spike gene of the severe acute respiratory syndrome coronavirus were demonstrated to be crucial for viral infection [37]. On the other hand, mutations observed in the neutralizing regions have been proposed as presenting a viral evolution, allowing escape from antibodies developed against vaccine or classical PEDV strains [38]. In this study, we characterized differences in epitope regions, high-specificity N-glycosylation and phosphorylation sites, between the vaccine and recent field strains. Various substitutions observed in the S (521, 553, 568, and 570), M (5), and N (123, 252, and 255) proteins may have affected the antigenicity, conferring the capacity for immune evasion in the PEDV field strains, and consequently, influencing the efficacy of the vaccines. In fact, despite vaccination, PEDV infections still spread rapidly and are commonly observed on PED-vaccinated farms in Japan [23]. Vaccination showed very low efficacy and could not mitigate the severe losses caused by PED [39]. Based on the antigenicity analysis of the vaccine and field strains, as well as the recent performance of the PED vaccines on the Japanese pig farms, we suggest that the development of novel vaccines based on reemerging PEDVs is necessary to control current PED outbreaks.

The first PED epidemic on farm 1 was a severe occurrence in May 2014 (Table 3). Afterward, obvious clinical symptoms and death caused by PED were not observed in pigs of the herd until February 2016 when the subsequent outbreak recurred with less severity than the first. Although the pigs did not show any obvious symptoms of PED, the presence of PEDV on this farm was detected in piglets several times by RT-PCR during the period from June 2014 to January 2016, according to the information supplied by veterinarians responsible for this farm. Phylogenetic analysis based on the sequences of the S and N genes showed that PEDV strains from the primary and secondary outbreaks formed monophyletic branches. They also shared with each other the highest sequence identity of the S, M, and N genes compared to other strains used in this study. Additionally, PED outbreaks were not observed on other farms within a 5 km radius of farm 1, and many strict measures for establishing a high level of security were implemented on this farm. Taken together, we speculated that PEDV strains causing the primary outbreak induced partial protective immunity but persisted in the herd for nearly two years. Subsequently, the persisted PEDVs caused the secondary epidemic. This is the first report of a two-year-long persistence of PEDV in a pig herd. It has been proposed that the popular circulation of PEDV variants with large deletions in the S gene is associated with the persistence of the PEDV on the infected farms in Japan [24]. However, we did not detect the presence of the large S deletion variants in the fecal and intestinal samples collected from this farm. Other factors, including the genetic variation of causative PEDV strains, may play a more important role in the disease recurrence.

On farms 2 and 3, PED first occurred in May 2014, despite all the sows receiving antepartum PEDV vaccinations in 2013. Afterward, the disease disappeared until early 2016 when the subsequent outbreaks recurred. There was no detection of the PEDV during the interval between these two outbreaks. Phylogenetic analysis based on the S and N genes revealed that the strains collected from the first outbreaks on farms 2 and 3 formed a monophyletic branch. Moreover, these two farms were located close to each other with a distance of 0.5 km (separated by a small mountain), and the outbreaks occurred at the same time. This suggests that the PEDV strains introduced onto these farms that induced the first outbreaks were derived from a common source of PEDV. Likewise, in the secondary outbreaks, the strains collected from these two farms also have the highest genetic identity to each other and formed minor monophyletic branches, as shown in the phylogenetic trees for the S and N genes. All the strains collected from the primary and recurrent outbreaks on these farms were closely clustered into the subclade PED-J2, together with other strains collected from other farms located in Miyazaki prefecture (Fig. 1). This data suggested that the PEDV strains causing the subsequent outbreaks on farms 2 and 3 evolved from PEDVs that had been circulating in the Miyazaki region since 2014. There are two possibilities for the origin of the strains that caused the subsequent outbreaks on farms 2 and 3. First, PEDVs may have persisted silently on these farms since 2014, evolving, and then causing the subsequent outbreaks when suitable conditions arrived in early 2016. Another possibility is that PEDVs may have persisted on other pig farms, circulating in the Miyazaki region since the 2014 PED occurrence, after which the PEDVs were introduced again onto farms 2 and 3, causing the secondary epidemics. More data is needed to elucidate exactly the origin of these PEDVs strains. The occurrence of PED in these herds has partially indicated the failure of using the current PEDV vaccines, which are based on old seed stock of classical Japanese strains. Besides, the repeated outbreaks in these herds indicate that the disease has developed to an endemic stage. These results could be used to elucidate the prevalence of PEDVs and to contribute to the prevention of PED in the region.

## Conclusions

In conclusion, our study revealed that Japanese field PEDVs causing PED outbreaks during 2013–2016 could be classified into three types, and these PEDVs might have been introduced from overseas at the same time or during similar periods. The disease has developed to an endemic stage in which the PEDV can persist for a long time in these herds or these local regions causing subsequent epidemics. The distant genetic relationships and various aa substitutions between the Japanese field PEDVs and vaccine strains may be responsible for the unsuccessful PED control in Japan. Our findings will be useful in understanding the origin and molecular epidemiology of the PEDV, and helping to develop measures for the control of the disease.

## Additional files

Additional file 1: **Table S1.** Highly-specific N-glycosylation sites in the spike protein of the vaccine and field strains. (DOCX 13 kb)

Additional file 2: **Figure S1.** Amino acid substitutions in the M and N proteins between the field and vaccine strains. **A**: Amino acid substitutions in the M protein. The field strain 14JM-236 has an aa substitution, S213 N, resulting in the loss of a predicted phosphorylation site. The substitution at aa 5 in the M protein of the field strains may result in gaining a high-specificity N-glycosylation site (NGSI) compared with the vaccine strain 96-P4C6. **B**: Amino acid substitutions in the N protein. Two antigenic epitopes (NEP-D4 and NEP-D6) are marked by red bars. Compared with the vaccine strains, the Japanese field strains had 9 aa changes, including K123 N, A142T, T145A, M216 V, R241K, H242L, K 252 R, N255S, Q397L. A high-specificity N-glycosylation site (NFSQ) was predicted to occur at position 123 and a phosphorylation site was predicted to occur at positions 142, 166, and 255. (JPEG 10446 kb)

Additional file 3: **Figure S2.** Aligment of deduced S amino acid sequences of Japanese PEDVs from primary and recurent outbreaks. The deduced S protein of 14JM-168 had 11 amino acid substitutions (T211I, V312 M, A477S, K566 N, D569Y, G612D, E626D, F635 L, S722 N, T777 M, G888D) compared to those of 16JM-334 and 16JM-339. Deduced S proteins of 14JM-179, 14JM-181, 14JM-199, and 14JM-200 had 13 amino acid substitutions (Y6H, T24A, A311T, F345 V, T367 N, L416F, H524L, K587R, V674F, S925A, S968A, N1009S, and I1067M) compared to those of 16JM-319 and 16JM-326. (PDF 371 kb)

## Abbreviations

aa: Amino acid
M: Membrane
N: Nucleocapsid
NA: North American
nt: Nucleotide
PED: Porcine epidemic diarrhea
PEDV: Porcine epidemic diarrhea virus
RT: Reverse transcription
S: Spike
