# Supplementary material for: Molecular characterization of US-like and Asian non-S INDEL strains of porcine epidemic diarrhea virus (PEDV) that circulated in Japan during 2013–2016 and PEDVs collected from recurrent outbreaks
Source: BMC Vet Res. 2018 Mar 14;14:96. doi: 10.1186/s12917-018-1409-0 (PMC5852955; doi:10.1186/s12917-018-1409-0)
Supplement: Supplementary file 1 — Table S1. Highly-specific N-glycosylation sites in the spike protein of the vaccine and field strains. (DOCX 13 kb) [file 12917_2018_1409_MOESM1_ESM.docx]

Table S1. Highly-specific N-glycosylation sites in the spike protein of the vaccine and field strains.

| N-glycosylation site | 96-P4C6 | P5V | G2b Japanese isolates | 14JM-140, 14JM-144 | 13JM-291 |
| --- | --- | --- | --- | --- | --- |
| 60 | ─ | ─ | NSTW | ─ | NSTW |
| 117 | ─ | NTSA | ─ | ─ | ─ |
| 119 | NATA | ─ | NATA | ─ | NATA |
| 132 | NKTL | NKTL | ─ | NKTL | ─ |
| 217 | NVTS | NVTS | NVTS | NVTS | NVTS |
| 325 | NDTS | ─ | NDTS | NDTS | ─ |
| 352 | NSSD | NSSD | NSSN | NSSD | NSSD |
| 382 | ─ | ─ | NSTV (only 14JM-242) | ─ | ─ |
| 515 | ─ | NITV | ─ | ─ | NITV |
| 557 | NVTN | NVTN | ─ (except 14JM-268) | ─ | ─ |
| 782 | NISI | NISI | NISI | NISI | NISI |
| 1233 | NLTS | ─ | ─ | ─ | ─ |
| 1250 | NKTL | NKTL | NKTL | NKTL | NKTL |
| 1262 | NRTG | ─ | NRTG | NRTG | NRTG |

─: no highly-specific N-glycosylation was predicted at the position.

Only 14JM-242 has a predicted N-glycosylation at site 382 as compared to other vaccine and field strains. The strain 14JM-268 (subgroup G2b) does not have a predicted glycosylation at site 557.
