# Supplementary figures and images for: Molecular characterization of US-like and Asian non-S INDEL strains of porcine epidemic diarrhea virus (PEDV) that circulated in Japan during 2013–2016 and PEDVs collected from recurrent outbreaks
Source: BMC Vet Res. 2018 Mar 14;14:96. doi: 10.1186/s12917-018-1409-0 (PMC5852955; doi:10.1186/s12917-018-1409-0)

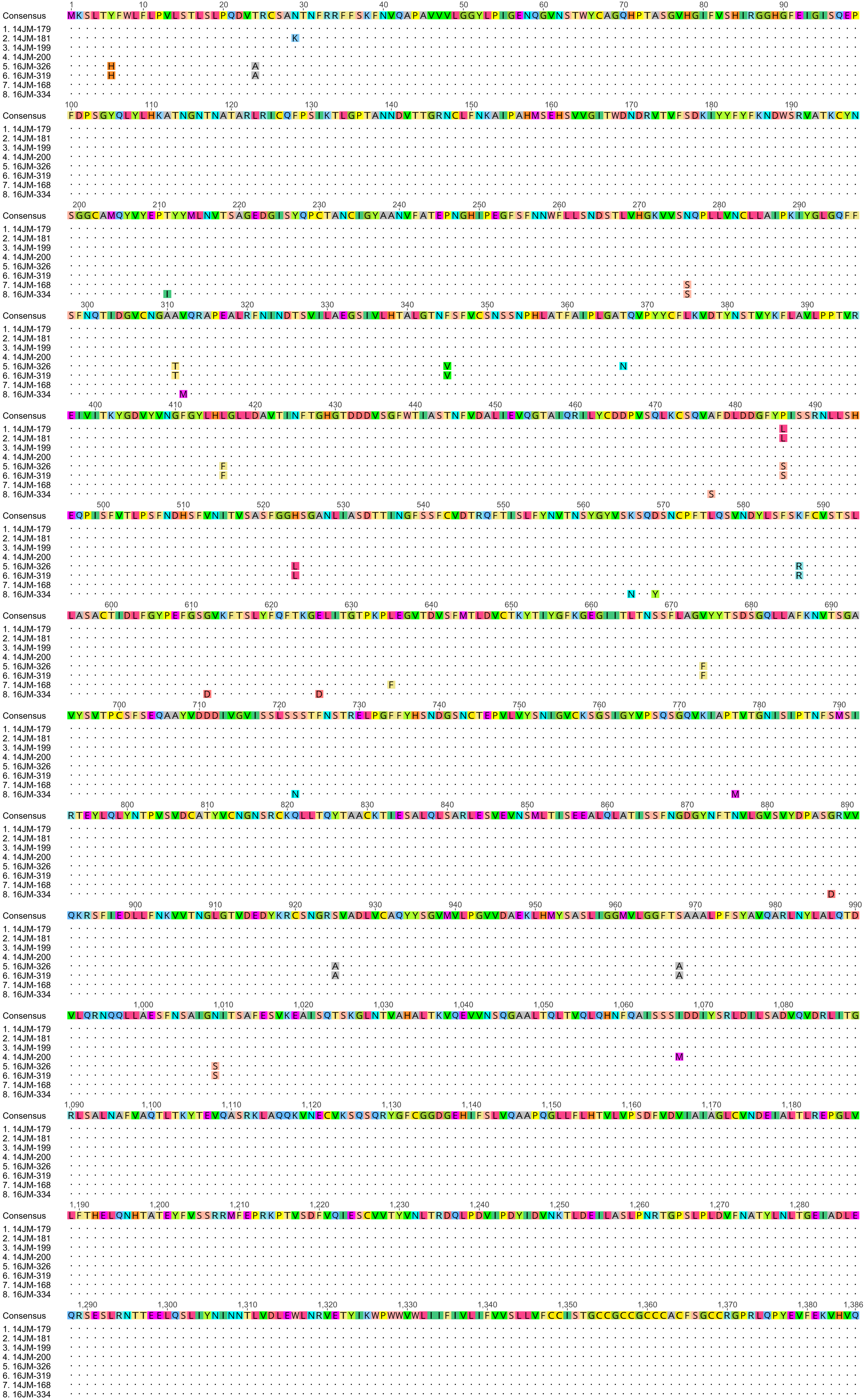

Supplement: Supplementary file 3 — Figure S2. Aligment of deduced S amino acid sequences of Japanese PEDVs from primary and recurent outbreaks. The deduced S protein of 14JM-168 had 11 amino acid substitutions (T211I, V312 M, A477S, K566 N, D569Y, G612D, E626D, F635 L, S722 N, T777 M, G888D) compared to those of 16JM-334 and 16JM-339. Deduced S proteins of 14JM-179, 14JM-181, 14JM-199, and 14JM-200 had 13 amino acid substitutions (Y6H, T24A, A311T, F345 V, T367 N, L416F, H524L, K587R, V674F, S925A, S968A, N1009S, and I1067M) compared to those of 16JM-319 and 16JM-326. (PDF 371 kb) [file 12917_2018_1409_MOESM3_ESM.pdf]
